# The Semi-Centennial of the Discovery of Anæsthesia

**Published:** 1894-07

**Authors:** 


### THE SEMI-CENTENNIAL OF THE DISCOVERY OF ANÆSTHESIA.

A VERY general interest in this matter has been awakened, and a number of local associations have decided to make the event of marked interest.

The editor of the Buffalo *Dental Practitioner and Advertiser*, Dr. Barrett, suggests "that every dental society in the land, as far as possible, appoint delegates to meet at Old Point Comfort at the time of the annual sessions of the American and Southern Dental Associations, there to agree upon some concerted plan of action."

It is hoped all associations will instruct their delegates to meet with this proposed body, and if not too late in the year arrange a definite course of procedure. If a national celebration of the event be deemed impracticable, a day might be decided upon for all the societies in the country to meet in their several localities and make the occasion not only impressive, but one of marked historical interest.

